# Dietary ginger (*Zingiber officinale*) enhances performance traits, biochemical and haematological indices of Turkey targeting mRNA gene expression

**DOI:** 10.1080/10495398.2024.2425656

**Published:** 2024-11-20

**Authors:** Eman A. Manaa, Mervat A. Abdel-Latif, Samya E. Ibraheim, Abdelaziz M. Sakr, Hanaa M. Ghanem, Rania M. Waheed, Ghadeer M. Albadrani, Mohamed M. Abdel-Daim, Amr El Zawily, Basant M. Shafik

**Affiliations:** a Animal and Poultry Production, Department of Animal Wealth Development, Faculty of Veterinary Medicine, Benha University, Toukh, Egypt; b Department of Nutrition and Veterinary Clinical Nutrition, Faculty of Veterinary Medicine, Damanhour University, Damanhour, Egypt; c Rabbit, Turkey and Water fowl Animal Production, Research Institute, Agricultural Research Center, Dokki, Giza, Egypt; d Biotechnology Department Research Institute, Agricultural Research Center, Dokki, Giza, Egypt; e Department of Animal Wealth Development, Faculty of Veterinary Medicine, Mansoura University, Mansoura, Egypt; f Department of Forensic Medicine and Toxicology, Faculty of Veterinary Medicine, Benha University, Toukh, Egypt; g Department of Biology, College of Science, Princess Nourah bint Abdulrahman University, Riyadh, Saudi Arabia; h Department of Pharmaceutical Sciences, Pharmacy Program, Batterjee Medical College, Jeddah, Saudi Arabia; i Pharmacology Department, Faculty of Veterinary Medicine, Suez Canal University, Ismailia, Egypt; j Department of Plant and Microbiology, Faculty of Science, Damanhour University, Damanhour, Egypt

**Keywords:** *Zingiber officinale*, bronze turkey, growth, blood parameters, growth genes

## Abstract

Ginger rich in polyphenols, possesses various biomedical properties. Researchers investigated the effects of dietary ginger supplementation on turkey performance traits, biochemical parameters, haematological parameters and mRNA gene expression. Ginger root powder was administered at different doses (0, 10, 20 and 40 g/kg) to the turkeys. Notably, the 20 g/kg group exhibited improved performance traits and a higher broiler production efficiency factor (BPEF). Importantly, ginger was found to be safe for turkeys based on serum indices. Furthermore, the expression of several growth-related genes, including growth hormone receptor (GHR), insulin-like growth factor 1 (IGF-1), adenine nucleotide translocase (ANT), cyclooxygenase 3 (COX-3) and uncoupling protein 3 (UCP-3), was upregulated in the 20 g/kg enhancing their growth performance and economic efficiency in addition to keeping their health status safe. Therefore, Ginger root powder can be supplemented for turkey at a concentration of 2% as the addition of ginger powder is a long-term process.

## Introduction

Since the COVID-19 pandemic, there has been increased interest in enhancing the production performance of grill chickens through the addition of efficient nutritional supplements to their feed.^1^ Ginger powder (*Zingiber officinale*) is among these ingredients. The growing global population necessitates increased food production and strategic planning to ensure food security. However, in poultry production, the prevalence of infections possesses significant economic challenges, contributes to antibiotic resistance and introduces additional environmental risks. According to regulations from the Food and Agriculture Organization and the United Nations, the use of natural additives as a substitute for antibiotic treatment is crucial. Natural alternatives were applied including prebiotics and probiotics,^2^^,^^3^ postbiotics^4–6^ and herbal extracts and byproducts.^7–10^ They have studied in poultry production and confirmed safe and effective additives if used in the optimal dose and acceptable duration.^11^^,^^12^ Moreover, polyphenols were shown as powerful heat stress mitigators in broilers and potential antioxidants.^13^^,^^14^

Ginger is a herbal plant that contains large amount of polyphenols which enhance the digestibility of food also the health of intestine which improve the feed efficiency.^15^ Furthermore, Ginger is immunomodulatory in lab animals and possesses antimicrobial qualities.^16^ Traditionally, ginger is used as a food spice with many biomedical properties.^17^^,^^18^ Ginger exhibits antioxidant properties that contribute to improved immunity in both livestock and birds.^19^ Not only does ginger powder offer antioxidant properties, but studies have shown that adding ginger powder to chicken diets improves broiler performance. Ginger’s antibacterial, anti-inflammatory, antioxidant, antiseptic, antiparasitic, and immunomodulatory qualities make it a valuable natural feed ingredient for chicken, particularly for broilers.^20^ Most people favour turkey meat as it is a good source of fatty acids, amino acids and proteins as well as essential vitamins and minerals. Lately, there has been a growing need to explore unconventional feeds and additives as a mean to mitigate the expenses associated with high-cost feed. Consequently, phytobiotics play an important role in enhancing performance and protecting against antibiotic resistance.

As of now, there have been no trials examining the effect of ginger on the expression of growth candidate genes in turkeys. Therefore, the current study aims to assess the influence of including *Zingiber officinale* extract in the diet on the productive performance, serum indices, and the expression of growth candidate genes in turkeys.

## Materials and methods

### Ethical approval

The study was accepted by the Committee of Local Experimental Animal Care, Damanhour University, Egypt, Faculty of Veterinary Medicine (DMU/VetMed-2023/049).

### Experimental design

Two hundreds and forty of 1-day Bronze turkey chicks were randomly divided into 4 groups of mixed sex with 60 chicks in each. Individual groups were subdivided into four replications (15 chick/ replication). The identification of chicks was by wing bands. The chicks were raised over a 20-week in a litter floor. Watering and feeding were freely applied. Standard conditions were applied for all birds. Light was used over 23 hours during the experiment. During the 1st two weeks, chicks were consumed a balanced basal diet, followed by the tested diets through the experiment (20^th^ week of age). Fresh ginger roots purchased from the Egyptian commercial market. The roots were turned into a powder that was added to the chicks’ food rations after being cleaned, cut, freeze-dried, ground, and milled into particle, its sizes were less than 1 mm and kept in air-tight pots at 25˚C until applying. The four groups (control, GRP10, GRP20 and GRP40) were fed with 0, 10, 20 and 40% ginger powder. The 4 groups (control, GRP10, GRP20 and GRP40) were fed with 0, 10, 20 and 40% ginger powder (obtained from a commercial market of medicinal plants, dried, milled into particle, its sizes were less than 1 mm and kept in air-tight pots at 25 ˚C until applying. The composition was chemically evaluated for crude protein (8.4), ether extract (5.8), ash (4.3), crude fiber (12.5), nitrogen-free extract (69) and dry matter (93) %, in that order.^21^ The makeup of control diet met the feeding standards of Bronze Turkey (Table 1).

**Table 1.** Composition and analysis of the brooding and growing diets (%, fresh basis).

Ingredients	Brooding (2–8 week)	Growing (8–20 week)
**Yellow Corn**	50.0	69.0
**Soybean meal (44% Crude Protein)**	39	20
**Fish meal (64% Crude Protein)**	10	10
**Di-Calcium Phosphate**	—	0.10
**Calcium carbonate**	0.40	0.30
**D-L meth.**	—	0.10
**L-Lys.**	0.10	0.15
Premix*	0.25	0.10
**Salt (Sodium chloride)**	0.25	0.25
**Total**	100	100
**Calculated chemical analysis, %**		
**Crude Protein**	27.5	20.87
**Metabolizable (kcal/kg)**	2830	3000
**CF**	3.9	2.99
**Ca**	0.79	0.72
**Avail. P**	0.43	0.42
**Lys.**	1.8	1.38
**Meth.**	0.78	0.8
**Meth.+ Cys.**	0.9	0.82

*Each 3 kilogram of premix contains the vitamin premix and trace mineral. The vitamin premix contributed the following: vitamin A 12,000,000 IU, vitamin D3 2,200,000 IU, vitamin E 10,000 mg, vitamin K3 2000 mg, vitamin B1 1000 mg, vitamin B2 4000 mg, vitamin B6 1000 mg, vitamin B12 10 mg, niacin 20,000 mg, biotin 50 mg, folic acid 1000 mg, pantothenic acid 10,000 mg. The trace mineral contributed the following: copper sulphate 10000 mg, potassium iodide 1000 mg, manganese oxide 55,000 mg, zinc oxide 50,000 mg, selenium 100 mg.

### Vaccination

The vaccination program was applied as the following: Hitchner one in water (Polimun-ND^®^, Bi-oTestLab, Kiev, Ukrain) at seven days. Inactivated H5N1 vaccine (MeFluvac^®^, MEVAC, Cairo, Egypt) at ten and thirty days; live Newcastle disease (Nobilis^®^ ND LaSota, MSD, Boxmeer, the Netherlands) vaccine in water at sixteen days; killed Newcastle disease (MEVAC-ND^®^, MEVAC, Cairo, Egypt) at forty five days; killed bivalent (H5N1 + ND) vaccine (Volvac^®^, Boehringer Ingelheim, Germany) at ninety days and lately vaccinated with killed Cholera vaccine (Servac^®^, Abbasia, Egypt). Subcutaneous injection was the route for all killed vaccines.

### Performance traits

Body weight was measured at one day old and monthly for twenty weeks. Average feed intake (AFI), feed conversion ratio (FCR), body weight gain (BWG), relative growth rate (RGR) and production efficiency factor (BPEF)^22^ were measured from the start to the end of the experiment.

### Sampling

Wing vein was used to collect blood (*n* = 16) (2 ml) at the age of 2^nd^ and 20^th^ week. About one millilitre was mixed with ethylene diamine tetra acetic acid to measure the haematological indices and 1 ml was centrifuged at 1435 × *g* for 14 minutes and the serum was separated for all biochemical indices using commercial kits. Sodium pentobarbital (I/V; 50 mg/kg) was used for birds euthanizing, necropsied immediately and 30 mg samples of liver (*n* = 16; 8 males and 8 females) were taken from each treatment and stored at −80 °C for gene expressions.

### Biochemical and haematological indices

Serum samples were assessed for biochemical parameters using commercial kits (Spinreact, Barcelona, Spain) with an Ultraviolet-visible spectrophotometer. The complete blood count was determined in whole blood samples using a Neubauer hemocytometer, diluted 1:200 with Natt and Herrick solution. Differential leukocyte count, haemoglobin (Hgb) concentration, and packed cell volume (PCV) were estimated as Campbel^23^ stated.

### RNA extraction and RT-PCR

In this study, RNA extraction was performed using Trizol^®^ (Invitrogen, Carlsbad, CA, USA). The quality and quantity of RNA were assessed using a spectrostar Nanodrop. The extracted RNA samples were stored at −80 °C. Subsequently, single-stranded cDNA was synthesized from the RNA (1000 ng) using high-capacity cDNA Reverse Transcription Kits (Applied Biosystems) and stored at −20 °C. Finally, gene expression analysis was conducted using Real-time polymerase chain reaction (PCR) to evaluate the expression of growth hormone receptor (GHR), insulin-like growth factor 1 (IGF-1), adenine nucleotide translocase (ANT), cyclooxygenase 3 (COX-3), and uncoupling protein 3 (UCP-3). Real-time polymerase chain reaction performed using the fluorescent dye SYBR Green (SYBR^®^ Green PCR Master Mix, Applied Biosystems, USA).

All reactions were conducted under uniform conditions and normalized using the ROX Reference Dye (Invitrogen, Carlsbad, CA, USA) to account for evaporation-related fluctuations in readings. Target gene primers were designed based on gene sequences deposited in GenBank (http://www.ncbi.nlm.nih.gov), with the endogenous housekeeping gene being β-actin (as indicated in Table 2). The changes in gene expression were computed using the comparative Ct method between the target and reference genes.^24^

**Table 2.** RT-PCR primers, candidate genes, and cycling conditions for RT-PCR.

Target Genes	Primer sequence (5′-3′)		Annealing temperature (°C)	Amplicon (bp)	Accession number and References
**GHR**	Forward	AACACAGATACCCAACAGCC	60 °C	145	KF957983.1^25^
	Reverse	AGAAGTCAGTGTTTGTCAGGG			
**IGF-I**	Forward	CACCTAAATCTGCACGCT	60 °C	140	NM_001004384.2^26^
	Reverse	CTTGTGGATGGCATGATCT			
**ANT**	Forward	TGTGGCTGGTGTGGTTTCCTA	60 °C	67	AB088686.1^27^
	Reverse	GCGTCCTGACTGCATCATCA			
**UCP-3**	Forward	GCAGCGGCAGATGAGCTT	60 °C	62	XM_015280964.2^28^
	Reverse	AGAGCTGCTTCACAGAGTCGTAGA			
**COX-3**	Forward	AGGATTCTATTTCACAGCCCTACAAG	60 °C	71	KC847746.1^29^
	Reverse	AGACGCTGTCAGCGATTGAGA			
**β-actin**	Forward	ACCCCAAAGCCAACAGA	60 °C	136	NM_205518.1^30^
	Reverse	CCAGAGTCCATCACAATACC			

### Statistical analysis

All data were statistically analysed using SPSS (IBM SPSS. 20^®^) utilizing the one-way ANOVA followed by Tukey’s multiple range tests at a significance level of *p* < 0.05. Additionally, RT-PCR data were analysed using GraphPad Prism 5 at *p* < 0.05.

## Results

All data resulted from investigating the effect of dietary ginger root powder supplementation at levels of 0, 10, 20 and 40 g/kg to turkey chicks on performance traits, serum indices and growth candidate genes are explained in the following sections:

### Performance traits

The impact of the ginger dietary inclusion on growth traits of turkeys during the study is shown in Table 3. The productive performance was improved in all ginger-supplemented groups especially in GRP20 and GRP40 throughout the experiment compared to control group, by 12.50, 13.25, 13.25 and 25.09% for FBW, ADG, FCR and BPEF, respectively; however, data of GRP10 was similar to the control one.

**Table 3.** Impact of dietary ginger inclusion in turkey diets on growth performance.

Items	*Control	Ginger supplementation			*p*-value
		GRP10	GRP20	GRP40	
**Initial weight, g**	251.70 ± 5.07	251.10 ± 3.25	250.70 ± 2.11	251.20 ± 2.61	0.99
**^1^FBW, g**	4870.4 ± 165.6^b^	4919.4 ± 151.6^b^	5479.0 ± 153.3^a^	5429.6 ± 137.0^a^	0.004
**^2^ADG, g**	32.98 ± 1.17^b^	33.34 ± 1.07^b^	37.35 ± 1.08^a^	36.99 ± 0.97^a^	0.004
**^3^RGR, %**	179.4 ± 0.69^b^	179.9 ± 0.52^b^	181.95 ± 0.43^a^	181.84 ± 0.42^a^	0.001
**^4^FI, g**	16698.76 ± 17.27^a^	16690.90 ± 10.03^b^	16682.1 ± 14.89^c^	16670.64 ± 13.20^d^	0.001
**^5^FCR**	3.85 ± 0.14^a^	3.76 ± 0.12^a^	3.34 ± 0.10^b^	3.34 ± 0.09^b^	0.002
**^6^BPEF**	142.58 ± 9.66^b^	144.30 ± 8.79^b^	178.36 ± 9.58^a^	173.89 ± 8.50^ab^	0.006

Values within the same row with different superscripts are significantly different (*p* < 0.05).

^1^Final body weight, ^2^Average daily gain, ^3^Relative growth rate, ^4^Feed intake, ^5^Feed conversion ratio and ^6^Broiler production efficiency factor.

*Each group consisted of 60 chicks with four replications (15 chicks/ replication).

### Biochemical and haematological indices

Tables 4 and 5 present the serum indices of turkeys at the 2^nd^ and 20^th^ weeks of age, respectively.

**Table 4.** Impact of dietary ginger inclusion in turkey diets on hematological and biochemical parameters at the 2^nd^ week of age.

Items	Control	Ginger supplementation			*p-value*
		GRP10	GRP20	GRP40	
**HB (g/dl)**	15.51 ± 0.64	16.90 ± 0.69	15.71 ± 0.57	14.58 ± 0.53	0.084
**HCT (%)**	46.49 ± 1.90	50.70 ± 2.08	47.14 ± 1.80	43.73 ± 1.19	0.083
**RBC (Cells/ul)**	4.77 ± 0.39	5.60±.0.23	5.20 ± 0.29	4.84 ± 0.25	0.120
**MCV (fl/cell)**	97.88 ± 0.45	90.36 ± 0.21	90.48 ± 0.20	90.45 ± 0.23	0.409
**MCH (pg/cell)**	32.48 ± 0.31	30.15 ± 0.16	30.20 ± 0.18	29.95 ± 0.23	0.456
**MCHC (g/dl)**	31.44 ± 0.05	33.31 ± 0.07	33.30 ± 0.02	33.08 ± 0.23	0.336
**PLT (× 10^3^/mm3)**	2743.8 ± 39.72	2737.5 ± 44.37	2918.8 ± 33.99	2818.8 ± 43.24	0.189
**WBC(× 10^3^/mm3)**	1606.2 ± 59.86	1400.0 ± 41.42	1675.0 ± 55.10	1475.0 ± 49.55	0.378
**GOT (U/L)**	269.50 ± 12.76	321.75 ± 17.87	292.25 ± 13.58	264.75 ± 11.28	0.192
**GPT (U/L)**	9.63 ± 0.45	11.63 ± 0.54	10.63 ± 0.42	9.63 ± 0.65	0.784
**Creatinine (mg/dl)**	0.31 ± 0.05	0.64 ± 0.07	0.26 ± 0.03	0.26 ± 0.03	0.435
**Urea (mg/dl)**	15.00 ± 0.64	20.38 ± 0.81	11.69 ± 0.89	10.71 ± 0.50	0.188
**BUN (mg/dl)**	7.01 ± 0.90	9.52 ± 0.95	5.46 ± 0.42	5.00 ± 0.34	0.188
**Triglyceride (mg/dl)**	118.75 ± 4.33	120.88 ± 4.27	87.63 ± 3.17	88.50 ± 2.82	0.084
**Cholesterol (mg/dl)**	147.62 ± 5.43	128.50 ± 4.27	131.75 ± 5.35	136.38 ± 6.32	0.655
**HDL (mg/dl)**	129.75 ± 4.18	122.00 ± 4.30	83.00 ± 3.02	72.62 ± 3.23	0.077
**LDL (mg/dl)**	61.08 ± 1.80	27.36 ± 1.08	59.08 ± 2.72	46.05 ± 2.67	0.127

Values within the same row with different superscripts are significantly different (p < 0.05).

HB: haemoglobin; HCT: Haematocrit; RBC: Red Blood Cells; MCV: Mean Cell Volume; MCH: Mean Cell Haemoglobin; MCHC: Mean Corpuscular Haemoglobin Concentration; PLT: Platelet; WBC: White Blood Cell; GOT: Glutamic Oxaloacetic Transaminase; GPT: Glutamic Pyruvic Transaminase; BUN: Blood Urea Nitrogen; HDL: High-Density Lipoprotein; LDL: Low-Density Lipoprotein.

**Table 5.** Impact Of ginger inclusion in Turkey diets on haematological and biochemical parameters at the 20^th^ week of age.

Items	Control	Ginger supplementation			*p-value*
		GRP10	GRP20	GRP40	
**HB (g/dl)**	16.80 ± 0.64	15.53 ± 1.01	14.43 ± 0.75	14.46 ± 0.84	0.160
**HCT (%)**	50.40 ± 1.92	46.58 ± 2.04	43.28 ± 2.25	43.39 ± 2.51	0.160
**RBC (Cells/ul)**	5.60 ± 0.21	5.17±.0.34	4.80 ± 0.25	4.82 ± 0.28	0.158
**MCV (fl/cell)**	90.04 ± 0.02	90.08 ± 0.02	90.10 ± 0.02	90.10 ± 0.02	0.068
**MCH (pg/cell)**	30.00 ± 0.00	30.00 ± 0.00	30.01 ± 0.01	30.01 ± 0.01	0.580
**MCHC (g/dl)**	33.30 ± 0.00	33.30 ± 0.00	33.30 ± 0.00	33.30 ± 0.00	1.00
**PLT (× 10^3^/mm3)**	2175.0 ± 33.96	1650.0 ± 32.61	2068.8 ± 34.46	1837.5 ± 47.78	0.212
**WBC(× 10^3^/mm3)**	1875.0 ± 62.94^a^	1400.0 ± 53.38^ab^	975.00 ± 30.07^b^	1325.0 ± 54.92^b^	0.002
**GOT (U/L)**	287.00 ± 10.95^ab^	264.75 ± 8.21^b^	251.62 ± 17.90^b^	317.25 ± 12.97^a^	0.007
**GPT (U/L)**	7.50 ± 0.65	6.75 ± 0.59	5.25 ± 0.25	8.25 ± 0.41	0.097
**Creatinine (mg/dl)**	0.19 ± 0.01	0.18 ± 0.02	0.19 ± 0.02	0.25 ± 0.03	0.101
**Urea (mg/dl)**	12.89 ± 0.49	11.08 ± 0.15	10.21 ± 0.26	11.63 ± 0.31	0.006
**BUN (mg/dl)**	5.77 ± 0.12	5.17 ± 0.07	5.43 ± 0.14	6.02 ± 0.41	0.006
**Triglyceride (mg/dl)**	72.13 ± 6.77	53.13 ± 5.70	51.75 ± 4.90	46.75 ± 4.41	0.196
**Cholesterol (mg/dl)**	132.00 ± 3.15	144.75 ± 6.86	142.50 ± 5.96	155.25 ± 8.77	0.456
**HDL (mg/dl)**	68.75 ± 4.03^b^	81.13 ± 3.20^ab^	83.63 ± 3.76^ab^	87.00 ± 5.01^a^	0.050
**LDL (mg/dl)**	58.90 ± 3.90	53.28 ± 1.99	46.68 ± 1.37	44.45 ± 2.26	0.691

Values within the same row with different superscripts are significantly different (*p* ≤ 0.05).

HB: haemoglobin; HCT: Haematocrit; RBC: Red Blood Cells; MCV: Mean Cell Volume; MCH: Mean Cell Haemoglobin; MCHC: Mean Corpuscular Haemoglobin Concentration; PLT: Platelet; WBC: White Blood Cell; GOT: Glutamic Oxaloacetic Transaminase; GPT: Glutamic Pyruvic Transaminase; BUN: Blood Urea Nitrogen; HDL: High-Density Lipoprotein; LDL: Low-Density Lipoprotein.

Different ginger root powder treatments resulted in minimal changes in haematological and biochemical indices. However, renal biomarkers, lipid profiles, and liver function (evidenced by low ALT levels) significantly improved in the ginger-treated group, especially with the GRP20 treatment, compared to the control group.

### Gene expression

**Figure 1.**
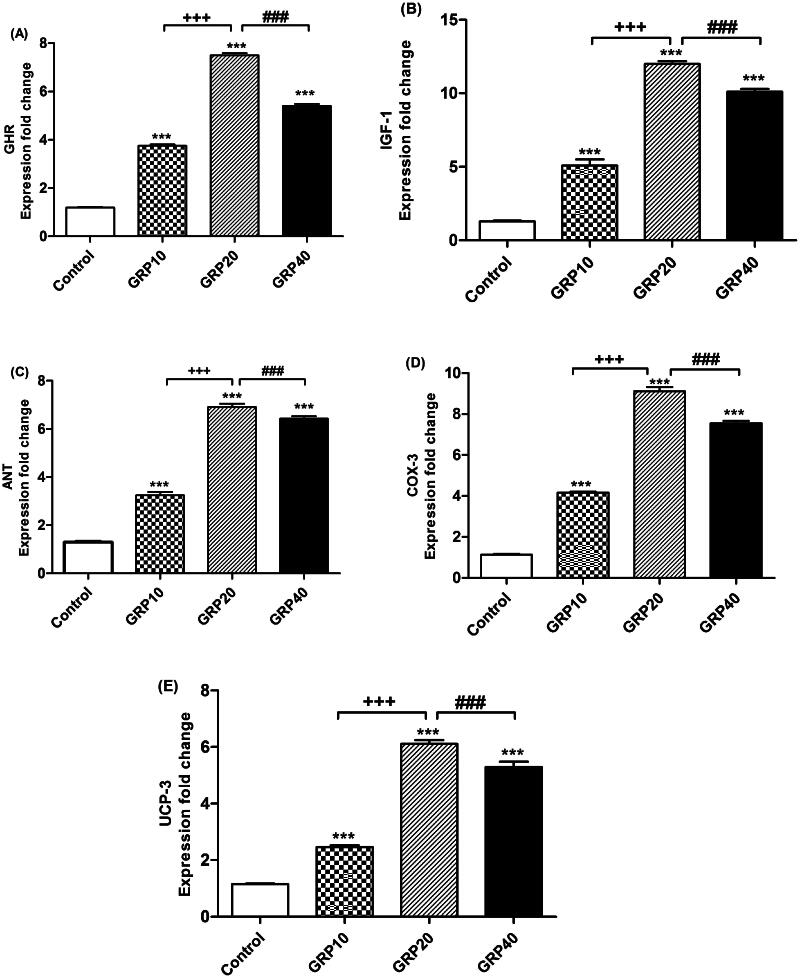
RT-PCR validation of the GHR (a), IGF-1 (B), ANT (C), COX-3 (D), and UCP-3 (E) genes. ***p < 0.001 *vs*. control. +++*p* < 0.001 *vs*. GRP10. ###*p* < 0.001 *vs*. GRP40. GRP10, birds fed 10 g/kg. GRP20, birds fed 20 g/kg. GRP40, birds fed 40 g/kg ginger.

In Fig. 1(A–E), we observed the expression patterns of growth candidate genes. Notably, the mRNA levels of several key genes-growth hormone receptor (GHR), insulin-like growth factor 1 (IGF-1), cyclooxygenase 3 (COX-3), adenine nucleotide translocase (ANT), and uncoupling protein 3 (UCP-3) were significantly higher (*p* < 0.001) in the ginger-treated groups compared to the non-supplemented group. Specifically, the expression was most pronounced in the GRP20, GRP40, and GRP10 treatments.

## Discussion

In the context of bird health and performance, antibiotics play a crucial role in preventing infections. However, it is equally imperative to explore natural alternatives to mitigate the risk of antibiotic resistance.^31^ *Zingiber officinale* contains significant levels of polyphenols, which have growth promoting and antibiotics like actions.^32^^,^^33^ Here, we are evaluating the various effects of *Zingiber officinale* as natural alternatives on performance, plasma constituents of turkeys during different stages of growth. Throughout the study, turkeys that received *Zingiber officinale* showed improvements in final body weight (fBwt), average daily gain (ADG), feed conversion ratio (FCR), and biological performance efficiency factor (BPEF). However, feed intake (FI) decreased with increasing ginger supplementation. These findings align with other studies that report the positive impacts of ginger in enhancing the production of turkey poults and chickens.^34^^,^^35^

The positive effects of ginger supplementation on turkeys’ growth performance, including enhanced feed efficiency and improved nutrient digestibility due to protease and lipase secretions, have been consistently observed.^36^^,^^37^ Several studies proved the best inclusion rate of ginger root powder which enhance the growth rate of broilers is up to 2%.^38^^,^^39^ In addition, reduced feed consumption was observed by Herawati & Marjuki and Al-Homidan ^40^^,^^41^ who found that supplementing ginger to a level up to 2 or 6% decreases the growth of broilers during the starter phase. As a result, the low growth rate of GRP40 may be attributed to the consequences of high doses due to its severe bitter taste.^42^ Ginger’s antioxidant properties improve turkey growth by protecting gut health. This enhances resistance to harmful pathogens that could disrupt digestion and nutrient absorption.^16^ Blood parameters are good indicators of turkey well-being. Although changes in blood parameters were not significant with *Zingiber officinale* treatments, there was a slight improvement in kidney and liver functions and lipid profiles. These findings proved that *Zingiber officinale* dietary inclusion is safe for turkey in different growth stages. At the same line, Shanoon *et al*.^43^, Hossain *et al*.^44^, Zhang *et al* ^45^ found no change either in kidney, liver, or hematological function biomarkers in broilers, while, Herve *et al*.^46^ approved their reduction. In addition, free radicals and lipid profile were decreased in supplemented quails.^34^ These results may be attributed to ginger’s anti-hypercholesterolemia and anti-lipidemic properties. Dietary ginger lowers total blood cholesterol by inhibiting hydroxylmethyl-glutaryl-coenzyme-A reductase.^47^

Growth candidate genes like growth hormone receptor (GHR), insulin like growth factor 1 (IGF-1), adenine nucleotide translocase (ANT), cyclooxygenase 3 (COX-3) and uncoupling protein 3 (UCP-3) were upregulated (*p* < 0.001) in *Zingiber officinale* treated groups especially for GRP20, which correlated with turkey’s enhanced growth performance under the current trial conditions. The selected genes were studied as efficient markers for improving growth of quails.^48^ Although there were insufficient studies investigating the impact of *Zingiber officinale* on the expression of growth-related candidate genes, it was observed that the anti-inflammatory candidate gene NF-kB was upregulated in birds treated with ginger. The upregulation of NF-kB was associated with the growth-candidate genes (e.g., IGF-1) regulating its expression as a health-related gene.^49^ Further research is imperative to investigate the effect of *Zingiber officinale* on the expression of growth candidate genes in turkey. Regardless to the current experiment; the upregulation of animals’ growth candidate genes hasn’t been confirmed as a result of herbal extracts supplementation.^50^ The enhanced growth and feed efficiency observed in the current study can be attributed to increased expression of the GHR gene in ginger-treated birds. This upregulation likely contributes to improved feed utilization, which results from the enhanced palatability of their diets.

## Conclusion

In summary, supplementing turkey feed with exogenous ginger root powder at concentrations of 2% and 4% improves growth performance (FBW, ADG, and FCR), economic efficiency (BPEF), and the expression of growth-candidate genes (GHR, IGF-1, ANT, COX-3 and UCP-3), while maintaining their health safe regarding haematological and biochemical parameters. So, t is recommended to use ginger root powder at a level of 2% as the addition of ginger powder is a long-term process.

## Data Availability

The data of this study are available from the corresponding authors, [MAA & EAM], upon reasonable request.

## References

[1] Hafez HM, Attia YA. Challenges to the poultry industry: current perspectives and strategic future after the COVID-19 outbreak. *Front Vet Sci*. 2020;7:516. 33005639 10.3389/fvets.2020.00516 PMC7479178

[2] Abdel-Latif MA, Abd El-Hack ME, Swelum AA, et al. Single and combined effects of Clostridium butyricum and Saccharomyces cerevisiae on growth indices, intestinal health, and immunity of broilers. *Animals*. 2018;8(10):184. 30347769 10.3390/ani8100184 PMC6210252

[3] Abou-Kassem DE, Elsadek MF, Abdel-Moneim AE, et al. Growth, carcass characteristics, meat quality, and microbial aspects of growing quail fed diets enriched with two different types of probiotics (Bacillus toyonensis and Bifidobacterium bifidum). *Poult Sci*. 2021;100(1):84–93. 33357710 10.1016/j.psj.2020.04.019 PMC7772674

[4] Abd El-Ghany WA, Abdel-Latif MA, Hosny F, et al. Comparative efficacy of postbiotic, probiotic, and antibiotic against necrotic enteritis in broiler chickens. *Poult Sci*. 2022;101(8):101988. 35809347 10.1016/j.psj.2022.101988 PMC9272375

[5] Ding J, He S, Xiong Y, Liu D, Dai S, Hu H. Effects of dietary supplementation of fumaric acid on growth performance, blood hematological and biochemical profile of broiler chickens exposed to chronic heat stress. *Braz J Poult Sci*. 2020;22(01):eRBCA–2019.

[6] Elbaz AM, Ibrahim NS, Shehata AM, Mohamed NG, Abdel-Moneim A-ME. Impact of multi-strain probiotic, citric acid, garlic powder or their combinations on performance, ileal histomorphometry, microbial enumeration and humoral immunity of broiler chickens. *Trop Anim Health Prod*. 2021;53(1):115. 33438056 10.1007/s11250-021-02554-0

[7] Abdel-Latif MA, El-Far AH, Elbestawy AR, Ghanem R, Mousa SA, Abd El-Hamid HS. Exogenous dietary lysozyme improves the growth performance and gut microbiota in broiler chickens targeting the antioxidant and non-specific immunity mRNA expression. *PLoS One*. 2017;12(10):e0185153. 29059196 10.1371/journal.pone.0185153 PMC5653193

[8] El-Hack A, Mohamed E, Alagawany M, Salah AS, Abdel-Latif MA, Farghly MF. Effects of dietary supplementation of zinc oxide and zinc methionine on layer performance, egg quality, and blood serum indices. *Biol Trace Elem Res*. 2018;184(2):456–462. 29081062 10.1007/s12011-017-1190-0

[9] Saeed M, Ayaşan T, Alagawany M, El-Hack M, Abdel-Latif M, Patra A. The role of ß-mannanase (Hemicell) in improving poultry productivity, health and environment. *Braz J Poult Sci*. 2019;21(03):eRBCA–2019.

[10] Saleh AA, El-Far AH, Abdel-Latif MA, Emam MA, Ghanem R, Abd El-Hamid HS. Exogenous dietary enzyme formulations improve growth performance of broiler chickens fed a low-energy diet targeting the intestinal nutrient transporter genes. *PLoS One*. 2018;13(5):e0198085. 29847558 10.1371/journal.pone.0198085 PMC5976200

[11] Manaa EA, Abdel-Latif MA, Ibraheim SE, et al. Impacts of Macleaya cordata on productive performance, expression of growth-related genes, hematological, and biochemical parameters in Turkey. *Front Vet Sci*. 2022;9:873951. 35903127 10.3389/fvets.2022.873951 PMC9325542

[12] Sugiharto S. Alleviation of heat stress in broiler chicken using turmeric (Curcuma longa)-a short review. *JABB*. 2020;8(3):215–222.

[13] Abdel-Latif MA, Elbestawy AR, El-Far AH, et al. Quercetin dietary supplementation advances growth performance, gut microbiota, and intestinal mrna expression genes in broiler chickens. *Animals*. 2021;11(8):2302. 34438756 10.3390/ani11082302 PMC8388376

[14] Saeed M, Naveed M, BiBi J, et al. The promising pharmacological effects and therapeutic/medicinal applications of Punica granatum L.(Pomegranate) as a functional food in humans and animals. *Recent Pat Inflamm Allergy Drug Discov*. 2018;12(1):24–38. 29473532 10.2174/1872213X12666180221154713

[15] Barazesh H, Boujar Pour M, Salari S, Abadi M. T. The effect of ginger powder on performance, carcass characteristics and blood parameters of broilers. *International Journal of Advanced Biological and Biomedical Research*. 2013;1(12):1645–1651.

[16] Dieumou F, Teguia A, Kuiate J, Tamokou J, Fonge N, Dongmo M. Effects of ginger (Zingiber officinale) and garlic (Allium sativum) essential oils on growth performance and gut microbial population of broiler chickens. *Livestock Research for Rural Development*. 2009;21(8):23–32.

[17] Phillips S, Ruggier R, Hutchinson S. Zingiber officinale (ginger)–an antiemetic for day case surgery. *Anaesthesia*. 1993;48(8):715–717. 8214465 10.1111/j.1365-2044.1993.tb07188.x

[18] Utpalendu J, Chattopadhyay RN, Badri PS. Preliminary studies on anti-inflammatory activity of Zingiber officinale Rosc., Vitex negundo Linn and Tinospora cordifolia (willid) Miers in albino rats. *Indian Journal of Pharmacology*. 1999;31(3):232.

[19] Bosisio E, Benelli C, Pirola O. Effect of the flavanolignans of Silybum marianum L. on lipid peroxidation in rat liver microsomes and freshly isolated hepatocytes. *Pharmacol Res*. 1992;25(2):147–154. 1635893 10.1016/1043-6618(92)91383-r

[20] Onu P. Evaluation of two herbal spices as feed additives for finisher broilers. *Bio Anim Husb*. 2010;26(5-6):383–392.

[21] Lee J, Durst R, Wrolstad R. AOAC official method 2005.02: total monomeric anthocyanin pigment content of fruit juices, beverages, natural colorants, and wines by the pH differential method. *Official Methods of Analysis of AOAC International*. 2005;1:2. 16385975

[22] Murugan M, Ragavan A. Broiler performance efficiency factor (bpef) in commercial broiler production facilities with special reference to climate. *Indian Veterinary Journal*. 2017;94(03):11–14.

[23] Campbel T. *Avian Hematology and Cytology, Iowa State University Press.* Ames, IOWA. 1995.

[24] Livak KJ, Schmittgen TD. Analysis of relative gene expression data using real-time quantitative PCR and the 2− ΔΔCT method. *Methods*. 2001;25(4):402–408. 11846609 10.1006/meth.2001.1262

[25] Huang H, Zhao Z, Li S, Liang Z, Li C, Wang Q. Pattern of GHR mRNA expression and body growth in the S2 line of sex-linked dwarf chickens. *Genet Mol Res*. 2016;15(4):1–7. 10.4238/gmr15047416 27819736

[26] Saneyasu T, Tsuchihashi T, Kitashiro A, et al. The IGF‐1/Akt/S6 pathway and expressions of glycolytic myosin heavy chain isoforms are upregulated in chicken skeletal muscle during the first week after hatching. *Anim Sci J*. 2017;88(11):1779–1787. 28594135 10.1111/asj.12847

[27] Toyomizu M, Ueda M, Sato S, Seki Y, Sato K, Akiba Y. Cold-induced mitochondrial uncoupling and expression of chicken UCP and ANT mRNA in chicken skeletal muscle. *FEBS Lett*. 2002;529(2-3):313–318. 12372620 10.1016/s0014-5793(02)03395-1

[28] Li Q, Xu Z, Liu L, et al. Effects of breeds and dietary protein levels on the growth performance, energy expenditure and expression of avUCP mRNA in chickens. *Mol Biol Rep*. 2013;40(4):2769–2779. 23430386 10.1007/s11033-012-2030-0

[29] Sun J, Zhong H, Chen SY, Yao YG, Liu YP. Association between MT-CO3 haplotypes and high-altitude adaptation in Tibetan chicken. *Gene*. 2013;529(1):131–137. 23850731 10.1016/j.gene.2013.06.075

[30] De Boever S, Vangestel C, De Backer P, Croubels S, Sys S. Identification and validation of housekeeping genes as internal control for gene expression in an intravenous LPS inflammation model in chickens. *Vet Immunol Immunopathol*. 2008;122(3-4):312–317. 18272235 10.1016/j.vetimm.2007.12.002

[31] Robinson K, Becker S, Xiao Y, et al. Differential impact of subtherapeutic antibiotics and ionophores on intestinal microbiota of broilers. *Microorganisms*. 2019;7(9):282. 31443457 10.3390/microorganisms7090282 PMC6780560

[32] Asghar A, Farooq M, Mian M, Khurshid A. Economics of broiler production in Mardan Division. *Journal of Rural Development and Administration*. 2000;32(3):56–65.

[33] An K, Zhao D, Wang Z, Wu J, Xu Y, Xiao G. Comparison of different drying methods on Chinese ginger (Zingiber officinale Roscoe): changes in volatiles, chemical profile, antioxidant properties, and microstructure. *Food Chem*. 2016;197 Pt B:1292–1300. 10.1016/j.foodchem.2015.11.033 26675871

[34] Daramola O, Jimoh O, Akinnate A. Herbal effects of ginger in turkey poults. *Nigerian Journal of Animal Science*. 2020;22(3):122–127.

[35] Habibi R, Sadeghi G, Karimi A. Effect of different concentrations of ginger root powder and its essential oil on growth performance, serum metabolites and antioxidant status in broiler chicks under heat stress. *Br Poult Sci*. 2014;55(2):228–237. 24697550 10.1080/00071668.2014.887830

[36] Zhao X, Yang Z, Yang W, Wang Y, Jiang S, Zhang G. Effects of ginger root (Zingiber officinale) on laying performance and antioxidant status of laying hens and on dietary oxidation stability. *Poult Sci*. 2011;90(8):1720–1727. 21753209 10.3382/ps.2010-01280

[37] Herawati O. The effect of red ginger as phytobiotic on body weight gain, feed conversion and internal organs condition of broiler. *International J of Poultry Science*. 2010;9(10):963–967.

[38] Onimisi P, Dafwang I, Omage J. Growth performance and water consumption pattern of broiler chicks fed graded levels of ginger waste meal. Journal of Agriculture. *Forestry and the Social Sciences*. 2005;3(2):113–119.

[39] Ademola S, Farinu G, Babatunde G. Serum lipid, growth and haematological parameters of broilers fed garlic, ginger and their mixtures. *World J Agric Sci*. 2009;5(1):99–104.

[40] Herawati M, Marjuki K. The effect of feeding red ginger (Zingiber officinale rosc) as phytobiotic on broiler slaughter weight and meat quality. *International J of Poultry Science*. 2011;10(12):983–986.

[41] Al-Homidan A. Efficacy of using different sources and levels of Allium sativum and Zingiber officinale on broiler chicks performance. *Saudi Journal of Biological Sciences*. 2005;12:96–102.

[42] Hosseini M. Comparison of using different level of black pepper with probiotic on performance and serum composition on broilers chickens. *Journal of Basic and Applied Scientific Research*. 2011;1(11):2425–2428.

[43] Shanoon AK, Jassim MS, Amin QH, Ezaddin IN. Effects of ginger (Zingiber officinale) oil on growth performance and microbial population of broiler Ross 308. *International J of Poultry Science*. 2012;11(9):589–593.

[44] Hossain MM, Cho SB, Kang D-K, Nguyen QT, Kim IH. Comparative effects of dietary herbal mixture or guanidinoacetic acid supplementation on growth performance, cecal microbiota, blood profile, excreta gas emission, and meat quality in Hanhyup-3-ho chicken. *Poult Sci*. 2024;103(4):103553. 38417333 10.1016/j.psj.2024.103553 PMC10907848

[45] Zhang Z, Rolando A, Kim I. Effects of benzoic acid, essential oils and Enterococcus faecium SF68 on growth performance, nutrient digestibility, blood profiles, faecal microbiota and faecal noxious gas emission in weanling pigs. *Journal of Applied Animal Research*. 2016;44(1):173–179.

[46] Herve T, Raphaël KJ, Ferdinand N, et al. Growth performance, serum biochemical profile, oxidative status, and fertility traits in male Japanese quail fed on ginger (Zingiber officinale, roscoe) essential oil. *Vet Med Int*. 2018;2018:7682060–7682068. 30050674 10.1155/2018/7682060 PMC6046138

[47] Al-Khalaifah H, Al-Nasser A, Al-Surrayai T, et al. Effect of ginger powder on production performance, antioxidant status, hematological parameters, digestibility, and plasma cholesterol content in broiler chickens. *Animals*. 2022;12(7):901. 35405889 10.3390/ani12070901 PMC8996832

[48] Manaa EA, El-Attrouny MM, Baloza SH, Ramadan SI. Selection for high body weight and its association with the expression profiles of somatotropic axis and mitochondrial genes in Japanese Quail. *Pakistan Veterinary Journal*. 2022;42(2):261–265.

[49] Wullaert A, Bonnet MC, Pasparakis M. NF-κB in the regulation of epithelial homeostasis and inflammation. *Cell Res*. 2011;21(1):146–158. 21151201 10.1038/cr.2010.175 PMC3193399

[50] Liu G, Wei Y, Wang Z, Wu D, Zhou A, Liu G. Effects of herbal extract supplementation on growth performance and insulin-like growth factor (IGF)-I system in finishing pigs. *J Anim Feed Sci*. 2008;17(4):538–547.

